# Individual and group reflection in lecture-based large groups lead to comparable learning success

**DOI:** 10.1007/s00508-025-02610-x

**Published:** 2025-09-08

**Authors:** Clemens Höbaus, Ralf Schmidmaier

**Affiliations:** 1 Division of Angiology, Department of Medicine II, Medical University of Vienna, Waehringer Guertel 18–20, 1090 Vienna, Austria; 2 Medizinische Klinik und Poliklinik IV, LMU-Klinikum München, München, Germany

**Keywords:** Undergraduate Medical education, Medical students, Memory retention, Reflection, Non-written reflection, Large group teaching, Lecture-based teaching

## Abstract

**Objective:**

The study aims to elucidate a possible effect of individual reflection (IR) or group reflection (GR) on short-term and long-term memory retention in a large group lecture-based environment.

**Methods:**

In this quasi-experimental study 656 medical students were enrolled to compare the impact of IR and GR directly after the lectures and 2 months later. Students were divided into two groups and given two different lectures using IR or GR in a cross-over fashion. Memory retention was estimated using multiple-choice questions analyzed by Student’s T‑test.

**Results:**

Memory retention was similar using both reflection methods at baseline (*p* = 0.867; *p* = 0.971) and follow-up (*p* = 0.885; *p* = 0.945). Interestingly, both reflection methods fostered good memory retention over time. Studentsʼ self-assessment initially favored GR as more beneficial, an effect that faded over time.

**Conclusion:**

Reflection might be a promising tool to foster memory retention in large lecture cohorts without added benefit from social interaction.

## Introduction

Large group lecture-based teaching has been the hallmark of medical education for centuries. This passive teaching method has been deemed to hinder knowledge acquisition in the medical education literature during the last decade [1, 2]. Several approaches to optimize the learners’ environment such as the ICAP (Interactive, Constructive, Active, Passive) framework have been developed [2]. According to the ICAP theory, student participation increases from passive via active to constructive and interactive and will lead to better memory retention [2]. A study involving 40–60 students replaced the lecture-based dermatology class with interactive elements and reported increased student satisfaction and better test results [1]. In contrast, another study including veterinary medical students showed similar outcomes between case-based learning (CBL) and conventional lecture-based education (*n* = 55 each) [3]. The use of audience response systems (ARS) is another approach to promote active participation, which has been tested in another lecture-based dermatology class [4]. The use of ARS increased student satisfaction but did not improve test scores [4]. A possible reason for those discrepancies might be that interactive teaching methods foster increased test scores in practical skill training [5]. At the same time, results were similar when theoretical content was evaluated according to a meta-analysis [5]. In line, the introduction of team-based learning (TBL) in a 2-week curriculum as a substitution of some lectures showed similar test results after the course [6]; however, the test scores were significantly higher over the long run in the TBL cohort after 1 and 2 years [6]. In contrast, a large cohort of pharmacy students used TBL (*n* = 222) in comparison to lectures (*n* = 147) and revealed better memory retention in the lectures cohort after 17 months [7].

The sample size in the evaluation of interactive teaching methods in comparison to lectures was mostly performed in small to medium-sized student cohorts (< 60 students); however, in the real-world setting, many lectures are given to a larger group of students and it is unclear how those results translate to larger groups and whether student interaction will foster the learning experience. According to the cognitive load theory, the learning environment can be optimized to reduce the individual extrinsic load a student faces by adapting the learning material and structuring the lecture into smaller parts [8]. Unfortunately, there is no consistent data on memory retention after a lecture and the whole concept of the learning pyramid has been scrutinized [9]. It seems thus insufficient to optimize the learning material to provide an adequate individual learning environment.

A possible solution might be the introduction of reflection phases to reduce time constraints and let students actively process the given information [10]. Reflection as a method has in various forms mainly been used in small groups to strengthen empathy and foster the group dynamics to increase learning performance [11]. Reflection is especially beneficial for students with prior thematic knowledge to integrate novel information and generate alternative solutions to a given problem [10]. The practical use of reflection in medical education showed an increased diagnostic competence in case-based teaching [12], while the use of reflection in simulation-based education did not increase students’ performance [13]. Reflection phases can be implemented according to the underlying teaching theory for individuals (learner-centered, cognitivism) or groups (team-centered, social cognitivism) [14]. In line, one report showed that student interaction via buzz groups was able to increase memory retention [15]. The goal of this study is to evaluate the possible impact of individual or group reflection in a large group (> 200 students) lecture based on short-term and long-term memory retention.

## Methods

The study was designed to include medical students in the fourth year of study of a 6-year medical curriculum at the Medical University of Vienna. The quasi-experimental study investigated the possible effect of 2 methods of reflection and was conducted during 2 mandatory lectures which included all registered 656 students. The study was approved by the institutional review board (*Clearingstelle Lehre*) and by the institutions’ data protection committee. Additional approval from the ethics committee was requested but not deemed necessary by the institution’s ethics committee. All data were interpreted according to the institutions’ good scientific practice guidelines and complied with the conditions set by the institutions’ data protection committee.

### Study design

The cohort of 656 medical students is divided into 2 groups by the central administration to reduce the group size and conform to space restrictions at the lecture halls. Students themselves can only choose a group (10 persons) for practical skills per semester but will be allocated to larger groups for theoretical medical education. This process resulted in 2 pseudo-randomized groups, including 285 and 371 students (groups A and B). The study was conducted in 2 successive medicine lectures involving 3 case-based topics 10–12 min per session. The lectures were distanced by a 15-min recreation period for the students. All lectures were given by the same teacher and used the same learning material.

### Lecture structure

The curriculum was screened for prior theoretical lectures on the involved topics to establish a knowledge baseline. The lectures were designed to integrate theoretical knowledge into clinical cases to cross-link the knowledge foundation with clinical information. Each topic started with a short fact-based case presentation followed by clinical as well as laboratory and/or imaging findings.

### Intervention

The three teaching topics per lecture were each immediately followed by a 2-min reflection phase. The reflection phase was either an individual reflection (IR) phase involving time to process the given information and make personal notes or a group reflection (GR) phase according to the think-pair-share concept [4, 16]. This process involved individual reflection, pairing the results with their neighbors, and sharing them with the whole group using an audience response system (ARS). All reflection phases were initiated using a prompt in German which translates to “What have I learned? What do I additionally need to think about?”.

**Fig. 1:**
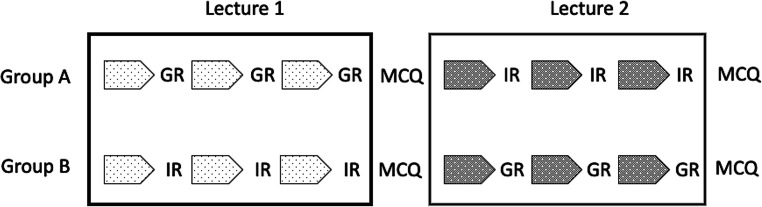
The cross-over study design depicts student cohorts A and B. *GR* group reflection, *IR* individual reflection, *MCQ* multiple choice test

The study was conducted using a cross-over design to minimize a possible selection bias by the small group selection process of students and to reduce a sequence bias in the use of the reflection phases. Students in group A were thus given lecture 1 (L1) using GR and lecture 2 (L2) using IR, while students in group B were given L1 using IR and L2 using GR as shown in Fig. 1. This redundancy allowed us to validate the results across two different topics of medicine.

### Data acquisition

Data were acquired to estimate short-term memory retention directly in the lecture hall using the ARS Slido® (Cisco Systems, Inc, San Jose, California, USA) licensed by the Medical University of Vienna. Students were given nine type A multiple-choice questions (MCQ) per lecture (three per topic) with a response time of 1 min after reading the given information. The results were not made available to the students. Students signed into the ARS system using a QR code without the need for identification. This allowed for anonymous reporting and web-based extraction of the data. In addition, the same system was used for the GR share part to the groups. This process provided a safe space for the students without the ability to identify individual pairs.

A voluntary follow-up was conducted 60 days after the lectures to collect data for long-term memory retention using the web-based Moodle platform of the Medical University of Vienna. Students were informed of this opportunity directly before this follow-up point in time during another mandatory lecture and given a QR code to participate. The students were invited to answer the same 18 MCQ (Cronbach’s alpha 0.82) given after the lecture in a shuffled format. The instructions were to answer the questions without auxiliary means and the answer time was automatically recorded by the systems. The time of 2 months was chosen to estimate long-term memory retention without interference with targeted learning for the upcoming examination set 1 month later. Additionally, this test provided the students with individual feedback before the study period. Students were asked for feedback regarding the two reflection methods and their sense of potential benefit at the end of the lecture (ARS) and during the voluntary follow-up.

### Statistics

The mean MCQ score for each lecture was used for comparison. Studentʼs unpaired T‑test was used to compare mean results between both types of reflection in L1 and L2 at baseline and follow-up. Unpaired analyses were conducted due to the use of ARS data at baseline, which does not identify individual students. Correlations between the number of cast votes (ARS) and the results of the MCQ questions were performed to rule out difficulty bias. An alpha of 0.05 was seen as significant. All statistical analyses were performed using IBM SPSS® V29. Figures were generated by SPSS or GraphPad Prism®.

## Results

The study population consists of medical students in their fourth year of medical education, which completes the theoretical part of the curriculum and is followed by clinical training in the subsequent remaining 2 years.

### Analyses of reflection methods—Short-term

**Fig. 2:**
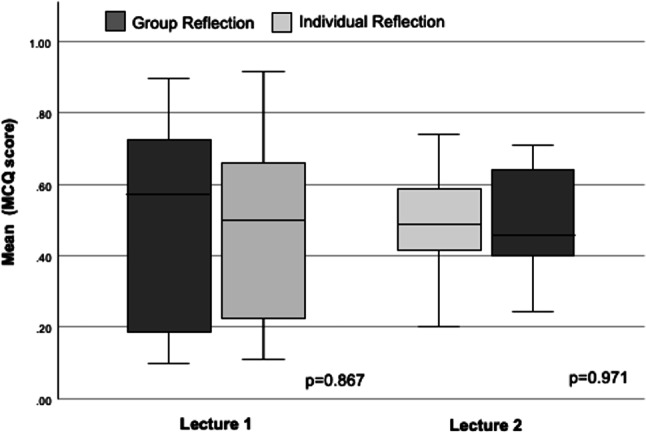
Box plot depicting MCQ score for both lectures at baseline categorized by group and individual reflection. MCQ scores were compared using the Student’s t‑test (*p*-value)

The ARS results between IR and GR measured at the end of the lecture were similar for L1 (0.47 ± 0.27 vs. 0.49 ± 0.31, *p* = 0.867) as well as L2 (0.49 ± 0.17 vs. 0.49 ± 0.17, *p* = 0.971) as depicted in Fig. 2. In detail, there was naturally a varying percentage of correct responses for each MCQ due to unequal difficulty levels; however, individual comparison of each MCQ between both reflection methods revealed no statistically significant differences. These results exclude relevant influences from the content and the time sequence of the lectures. Interestingly, there was no correlation between the number of cast votes (ARS) and the individual difficulty level of the MCQ (GR *p* = 0.508, IR *p* = 0.781). Furthermore, participation in the “share” part of the GR was similar between groups A and B with a participation maximum of 40–47%.

### Analyses of reflection methods—Long-term

**Fig. 3:**
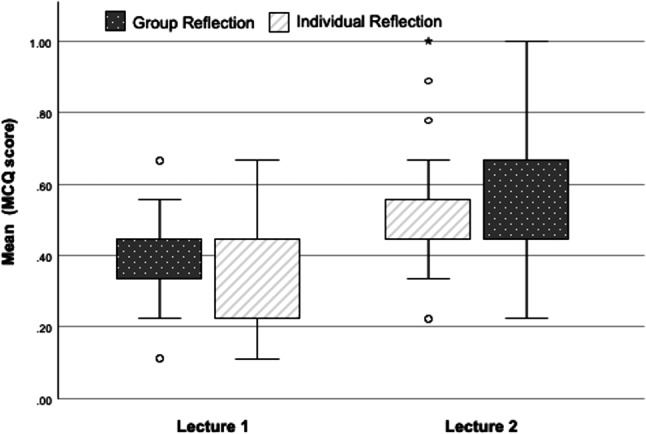
Box plot depicting MCQ score for both lectures at follow-up categorized by group and individual reflection. MCQ scores were compared using the Student’s t‑test (*p*-value)

In the optional long-term evaluation 115 students participated with complete test results. The results of the MCQ Moodle test showed similar results between both reflection methods for L1 (0.40 ± 0.13 vs. 0.40 ± 0.15 *p* = 0.885) and L2 (0.51 ± 0.17 vs. 0.50 ± 0.17, *p* = 0.945) as depicted in Fig. 3. These results were consistent across all 18 MCQs. Students needed 17 ± 11 min to answer the MCQ test excluding 19 students with an exam response time of over 60 min. Interestingly, those students’ responses were comparable to the overall cohort but probably interrupted the test before forwarding it. The mean test time of roughly a minute is comparable to the response time given during the lecture.

### Memory retention

**Fig. 4:**
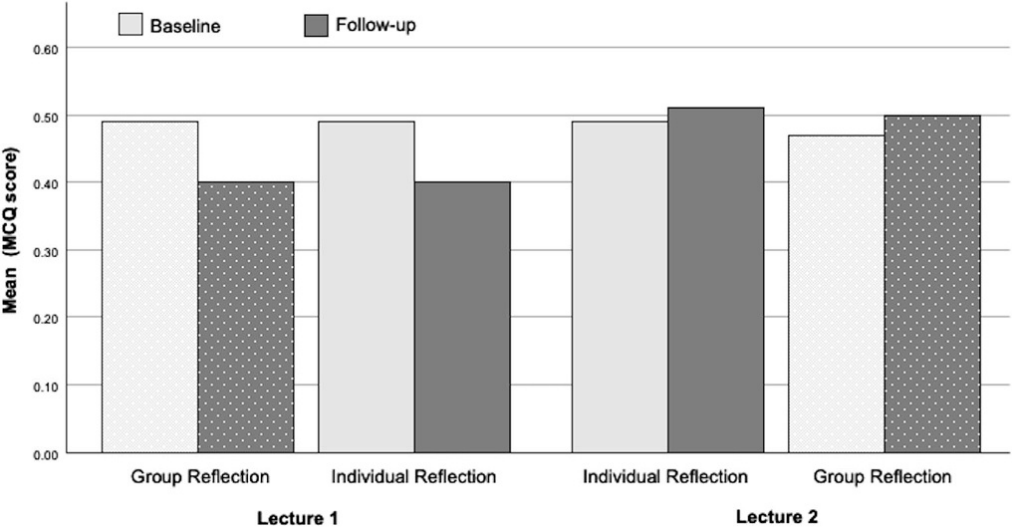
Memory retention at baseline and follow-up for both lectures by reflection method

Students exhibited good memory retention with similar results for IR and GR after 2 months. In detail, for L1 memory retention was reduced by 14% for IR and 18% for GR at follow-up, while for L2 memory retention was stable with a slight increase of 4% for IR and 2% for GR as depicted in Fig. 4. Student responses were analyzed to determine a possible cause for the discrepancy between L1 and L2 results. Two students scored 100% correct answers as outlier on L2 (whisker Fig. 3) while only scoring 25% and 56% correct on L1 in a mixed MCQ test rendering the use of auxiliary means as cause of the discrepancy unlikely.

### Self-assessment of students

**Fig. 5:**
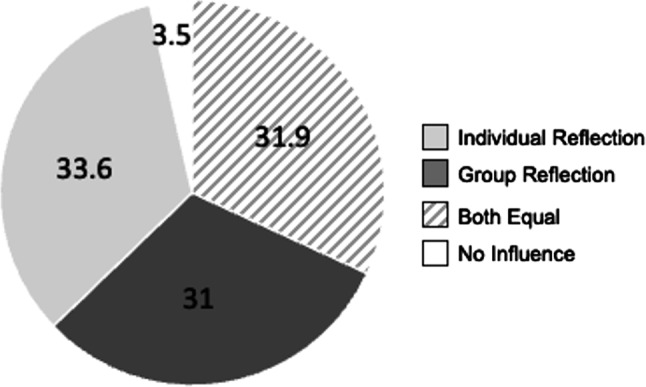
Student self-assessment of probable influence on memory retention by reflection method in %

Of the students 72% reported that the use of reflection will have an impact on memory retention. There was a preference for GR with 69% during the response in the lecture hall. Over time those results shifted to a nearly equal number of students thinking that individual or group reflection would be better for the study results, while one third regarded both reflection methods as equal as depicted in Fig. 5.

## Discussion

The findings of the study highlight that active engagement of students in lectures ensures good memory retention even in large groups, as compared to the published literature [9]. Interestingly, the method of individual reflection or group reflection with the assistance of an audience response system yielded similar results.

One reason might be that in a large group setting, no group-forming process is possible in a short time frame and the group reflection corresponds to a summary of two IRs; however, the sitting pattern in a large lecture hall is not random [17] and roughly 37.5% of students choose to sit next to a friend [18], which would foster a safe environment for group reflection [19]. This effect might further bias the results of the MCQ delivered through the ARS system as friends might consult each other on the correct answer. As the sitting pattern is presumably the same in both lectures, these effects apply equally to the IR and GR processes; however, theoretically, a pronounced effect could be seen in the IR group as this collaboration would be similar to the pair-process and overestimate memory retention in the IR group. Another reason might be that the allotted time frame was adequate for an IR but too short for a more complex GR process; however, through the “share” process in the GR, even students who were not actively engaging in the process would be presented with an additional visual stimulus by the group results. Furthermore, the ARS highlights responses that are given several times, which would further focus attention on recurrent answers of the group reflection process. Qualitative analyses of the GR results verified that the students acknowledged the main learning points of the presented material. The comparator IR is harder to evaluate as students were only taking notes for themselves. This study design was chosen to allow free association with the individual’s previous knowledge and integration of the new information. The requirement to submit the results of the IR for review would be quite similar to a 1-min paper, a method that is intended to evaluate the focus of the students on the teaching topic and raise awareness of possible misconceptions [20]; however, such an approach might lead the students to submit desired responses and hinder their personal learning experience, an effect that could also happen in the share process of the GR. Unfortunately, a comparison of both reflection methods to the same lecture without reflection is missing due to the challenge that the plain lecture would reduce teaching time by 6 min per lecture. Alternatively, the using of non-topic-related assignments to fill the void could distract studentsʼ attention and artificially reduce the memory retention for the control group.

Interestingly, memory retention declines early within 1 week after studying [21], thus long memory retention in both reflection groups was remarkably good. It would be expected that the memory retention results of both interactive and lecture-based formats will decline over time, with a lower retention rate at the end of the lecture [22]. The good recall after 2 months might be influenced by the MCQ test at the end of the lecture as testing after studying increases memory retention [21] and in fact-based lectures, active participation seems to play a minor role in maintaining long-term memory [7]; however, the given lectures were case-based which boosts memory retention at least in students using a computer simulation program [23]. The initial similar effects of both reflection methods underscore good short-term memory retention due to the structure of the lecture into time brackets and the use of active elements. Interestingly, even if short-term memory effects would have been shown they ameliorate over time [24], which makes it harder to evaluate long-term effects after a singular intervention. Furthermore, students were encouraged to think about their knowledge gaps at every single reflection prompt, which could foster students’ engagement with the topics after the lecture and thus increase memory retention in both groups through repeated study periods [21].

Students preferred GR as a method to increase their memory retention after the lecture but this preference faded over time. A Korean study showed that group cohesion increases in medical students over the study years and fosters learning [25]; however, this study included only 106 students and might thus not be transferable to a larger cohort. In a European cohort, students were randomized into 50-person cohorts with a high frequency of interaction which fostered group cohesion and psychological safety and restricted informal learning to those cohorts over time [26]. These effects might play a role in the immediate perception of the group process after the lecture taking factors such as sitting patterns into account and that all students were already enrolled for 4 years into the curriculum.

The study design proposed several limitations, such as that all students of the fourth study year were included during a mandatory teaching session. The motivation of the individual student to engage with the topic will vary. The lectures were based on already taught theoretical knowledge but no baseline knowledge testing was used to verify differences between the individual students. Theoretically, due to the central student allocation, this effect should be mitigated. Still, students with more interest in the topic or higher motivation to achieve a better grade on the examinations might be inclined to participate in the lecture. Concurrently, those are the students who have a higher likelihood to engage in the initial reflection processes and the follow-up. In line, the study showed no discrepancies in the proportional participation of the Moodle test between both groups. Furthermore, conducting the follow-up as an online examination is prone to manipulation but as the student’s interaction time with the test is comparable to the initial onsite test time it is unlikely that a relevant number of students used external means to change the MCQ scores as the tests were used as feedback by the students for the upcoming examination learning phase. Another limitation is that only 115 students provided a completed MCQ test at the follow-up which might bias the results; however, even at the initial lecture the response rate for each MCQ was varied and thus only a part of the attending students were engaging during the MCQ test. As the ARS does not track individual responses over several answers the study cannot evaluate if some students were engaged the whole time while others might only be interested in certain parts of the lecture and the corresponding MCQ. Students taking part in the optional MCQ test might be students with either a genuine interest in the topic or those who identified the relevance as preparation for the upcoming examinations at the end of the semester. This effect could overestimate the memory retention of the whole cohort; however, retesting students without intrinsic motivation might lead to arbitrary test scores, while those who participated did not exhibit unusually short answer times.

In summary, both reflection methods seem to provide the potential to increase memory retention over time in large lecture cohorts; however, it is unclear if the method itself or the introduction of individual self-learning phases into the lecture provided this effect. Interestingly, social interaction did not additionally foster memory retention. Thus, further evaluation of reflection in a tighter controlled learning environment is needed.

## References

[1] Ochsendorf FR, Boehncke WH, Sommerlad M, Kaufmann R. Interactive large-group teaching in a dermatology course. Med Teach. 2006;28(8):697–701. 17594580 10.1080/01421590601034241

[2] Chi MTH, Adams J, Bogusch EB, Bruchok C, Kang S, Lancaster M, et al. Translating the ICAP Theory of Cognitive Engagement Into Practice. Cogn Sci. 2018. 10.1111/cogs.12626 29954048

[3] Grauer GF, Forrester SD, Shuman C, Sanderson MW. Comparison of student performance after lecture-based and case-based/problem-based teaching in a large group. J Vet Med Educ. 2008;35(2):310–7. 18723821 10.3138/jvme.35.2.310

[4] Ivy KS, Larson AR. Incorporating engaged learning into medical student large group sessions. MedEdPublish. 2016;2017(6):120. 10.15694/mep.2017.000120 PMC10885274 38406424

[5] Zeng HL, Chen DX, Li Q, Wang XY. Effects of seminar teaching method versus lecture-based learning in medical education: A meta-analysis of randomized controlled trials. Med Teach. 2020;42(12):1343–9. 32795244 10.1080/0142159X.2020.1805100

[6] Ozgonul L, Alimoglu MK. Comparison of lecture and team-based learning in medical ethics education. Nurs Ethics. 2019;26(3):903–13. 28946799 10.1177/0969733017731916

[7] Taglieri C, Schnee D, Dvorkin CL, Zaiken K, Mistry A, Nigro S, et al. Comparison of long-term knowledge retention in lecture-based versus flipped team-based learning course delivery. Curr Pharm Teach Learn. 2017;9(3):391–7. 29233276 10.1016/j.cptl.2017.01.007

[8] van Merrienboer JJ, Sweller J. Cognitive load theory in health professional education: design principles and strategies. Med Educ. 2010;44(1):85–93. 20078759 10.1111/j.1365-2923.2009.03498.x

[9] Masters K. Edgar Dale’s Pyramid of Learning in medical education: Further expansion of the myth. Med Educ. 2020;54(1):22–32. 31576610 10.1111/medu.13813

[10] Chernikova O, Heitzmann N, Fink MC, Timothy V, Seidel T, Fischer F, et al. Facilitating Diagnostic Competences in Higher Education—a Meta-Analysis in Medical and Teacher Education. Educ Psychol Rev. 2020;32(1):157–96.

[11] Winkel AF, Yingling S, Jones AA, Nicholson J. Reflection as a Learning Tool in Graduate Medical Education: A Systematic Review. J Grad Med Educ. 2017;9(4):430–9. 28824754 10.4300/JGME-D-16-00500.1 PMC5559236

[12] Mamede S, van Gog T, Moura AS, de Faria RM, Peixoto JM, Rikers RM, et al. Reflection as a strategy to foster medical students’ acquisition of diagnostic competence. Med Educ. 2012;46(5):464–72. 22515754 10.1111/j.1365-2923.2012.04217.x

[13] Fink MC, Heitzmann N, Siebeck M, Fischer F, Fischer MR. Learning to diagnose accurately through virtual patients: do reflection phases have an added benefit? BMC Med Educ. 2021;21(1):523. 34620156 10.1186/s12909-021-02937-9 PMC8497044

[14] Hargreaves, K. Reflection in Medical Education. Journal of University Teaching and Learning Practice. 2016;13(2).

[15] Romeike BFM, Fischer M. Buzz groups facilitate collaborative learning and improve histopathological competencies of students. Clin Neuropathol. 2019;38(6):285–93. 31296286 10.5414/NP301195

[16] Guenther AR, Abbott CM. Think-Pair-Share: Promoting Equitable Participation and In-Depth Discussion. PRiMER. 2024;8:7. 38406233 10.22454/PRiMER.2024.444143 PMC10887392

[17] Benedict ME, Hoag J. Seating Location in Large Lectures: Are Seating Preferences or Location Related to Course Performance? J Econ Educ. 2004;35(3):215–31.

[18] Smith DP, Hoare A, Lacey MM. Who goes where? The importance of peer groups on attainment and the student use of the lecture theatre teaching space. Febs Open Bio. 2018;8(9):1368–78. 30186739 10.1002/2211-5463.12494 PMC6120247

[19] Phua GLG, Owyong JLJ, Leong ITY, Goh S, Somasundaram N, Poon EYL, et al. A systematic scoping review of group reflection in medical education. BMC Med Educ. 2024;24(1):398. 38600515 10.1186/s12909-024-05203-w PMC11007913

[20] Bartlett M, Morrow KA. Method for Assessing Course Knowledge in a Large Classroom Environment: An Improved Version of the Minute Paper. Am J Pharm Educ. 2001;65:264–7.

[21] Roediger HL, Karpicke JD. Test-enhanced learning: taking memory tests improves long-term retention. Psychol Sci. 2006;17(3):249–55. 16507066 10.1111/j.1467-9280.2006.01693.x

[22] Pamarthi V, Grimm L, Johnson K, Maxfield C. Hybrid Interactive and Didactic Teaching Format Improves Resident Retention and Attention Compared to Traditional Lectures. Acad Radiol. 2019;26(9):1269–73. 31085099 10.1016/j.acra.2019.02.018

[23] Subramanian A, Timberlake M, Mittakanti H, Lara M, Brandt ML. Novel educational approach for medical students: improved retention rates using interactive medical software compared with traditional lecture-based format. J Surg Educ. 2012;69(2):253–6. 22365876 10.1016/j.jsurg.2011.12.007

[24] Schmidmaier R, Ebersbach R, Schiller M, Hege I, Holzer M, Fischer MR. Using electronic flashcards to promote learning in medical students: retesting versus restudying. Med Educ. 2011;45(11):1101–10. 21988625 10.1111/j.1365-2923.2011.04043.x

[25] Kim S, Yang EB. Does group cohesion foster self-directed learning for medical students? A longitudinal study. BMC Med Educ. 2020;20(1):55. 32085775 10.1186/s12909-020-1962-7 PMC7035647

[26] Hommes J, Arah OA, de Grave W, Schuwirth LW, Scherpbier AJ, Bos GM. Medical students perceive better group learning processes when large classes are made to seem small. Plos One. 2014;9(4):e93328. 24736272 10.1371/journal.pone.0093328 PMC3988014

